# Detection of Iliopsoas Hematoma by Retrospective Radiography in Veno-Venous Extracorporeal Membrane Oxygenation for a COVID-19 Patient

**DOI:** 10.7759/cureus.22571

**Published:** 2022-02-24

**Authors:** Soichi Ohta, Kohei Takahashi, Masayuki Iwashita, Takeru Abe, Ichiro Takeuchi

**Affiliations:** 1 Advanced Critical Care and Emergency Center, Yokohama City University Medical Center, Yokohama, JPN

**Keywords:** intensive care, hemorrhage, extracorporeal membrane oxygenation, abdominal radiography, covid-19

## Abstract

In coronavirus disease 2019 (COVID-19), veno-venous extracorporeal membrane oxygenation (VV-ECMO) is used to manage respiratory distress. This study’s key clinical question was whether COVID-19 could be complicated by hemorrhagic and thrombotic events, such as iliopsoas hematoma (IPH), during the management of ECMO and the method to quickly and effectively detect IPH. A 52-year-old man with fever and dyspnea was diagnosed with COVID-19 pneumonia. He warranted VV-ECMO management on day 9, which was successfully tapered off on day 18. On day 20, computed tomography revealed a unilateral iliopsoas hematoma that was successfully managed with conservative care. However, a retrospective review of abdominal radiography performed on day 14 revealed a positive left psoas sign. When managing severe COVID-19 patients with VV-ECMO, cautious anticoagulative care and abdominal X-ray findings are warranted when considering the diagnosis of iliopsoas hematoma, including circulatory instability, anemia, and pain associated with limb movement.

## Introduction

Coronavirus disease 2019 (COVID-19) has been shown to be accompanied by microvascular problems such as lung capillary obstruction and macrovascular thromboembolism [1]. During the pandemic, veno-venous extracorporeal membrane oxygenation (VV-ECMO) has been used to manage respiratory distress. Under ECMO management, massive hemorrhage or abdominal compartment syndrome might warrant interventional radiology or surgical intervention [2]. In addition, we previously reported a high occurrence of iliopsoas hematoma (IPH) in Japanese patients [3]. Early detection of IPH is critical to the patient’s outcome. Here, we report the detection of IPH by radiography and conservative anticoagulation in a patient with severe COVID-19 during ECMO management.

## Case presentation

**Figure 1:**
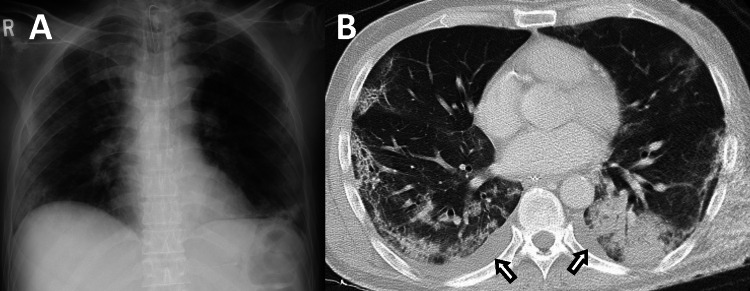
Chest X-ray and computed tomography on day 4 (A) Chest X-ray on day 4 showed bilateral peripheral consolidation. (B) Chest computed tomography on the same day also showed peripheral lung consolidation predominant on dorsal distribution. Pleural effusions were also seen (arrow).

This case involved a 52-year-old Japanese man with a history of type 1 diabetes mellitus (DM) and a smoking habit. The patient arrived at the hospital with initial complaints, including fever, cough, and shortness of breath that had persisted for four days. He reported no apparent contact with symptomatic patients or traveling overseas. The primary physical examination findings were: respiratory rate of 30 breaths/min, oxygen saturation (SpO2) of 97% (6 L/min oxygen via a facemask), heart rate of 135 bpm, blood pressure of 157/30 mmHg, and body temperature of 37.1°C. Chest radiography and computed tomography (CT) showed bilateral consolidation (Figure 1). An upper airway sample sent for polymerase chain reaction (PCR)-based testing for severe acute respiratory syndrome coronavirus 2 (SARS-CoV-2) was positive. A diagnosis of COVID-19 severe pneumonia was established, and he was transferred to our hospital for intensive care four days after the onset of symptoms.

The strong effort needed for breathing prompted us to intubate and establish mechanical ventilation support. We provided treatment that could be effective against COVID-19, including favipiravir and ciclesonide. On day 6, we switched favipiravir to remdesivir, a different antiviral agent that became available. Despite aggressive analgesia and sedation, the extreme effort required for the patient to breathe was unmanageable. On day 9, worsening respiratory acidosis, despite continuous paralysis, prompted us to establish VV-ECMO.

During ECMO management, anticoagulation therapy using unfractionated heparin continued to maintain active partial thrombin time (aPTT) levels within a range of 40-60 seconds. On day 11, prolonged fever led physicians to suspect catheter-related bloodstream infections, and we removed the central venous catheter. As lung consolidation on chest radiography and the patient's breathing effort showed gradual improvement, we terminated ECMO support on day 18. Afterward, we carefully tapered the treatment with sedatives, and the patient underwent respiratory rehabilitation and weaning from mechanical ventilation. We converted heparin administration from a continuous intravenous to a subcutaneous route for venothromboembolism prophylaxis.

**Figure 2:**
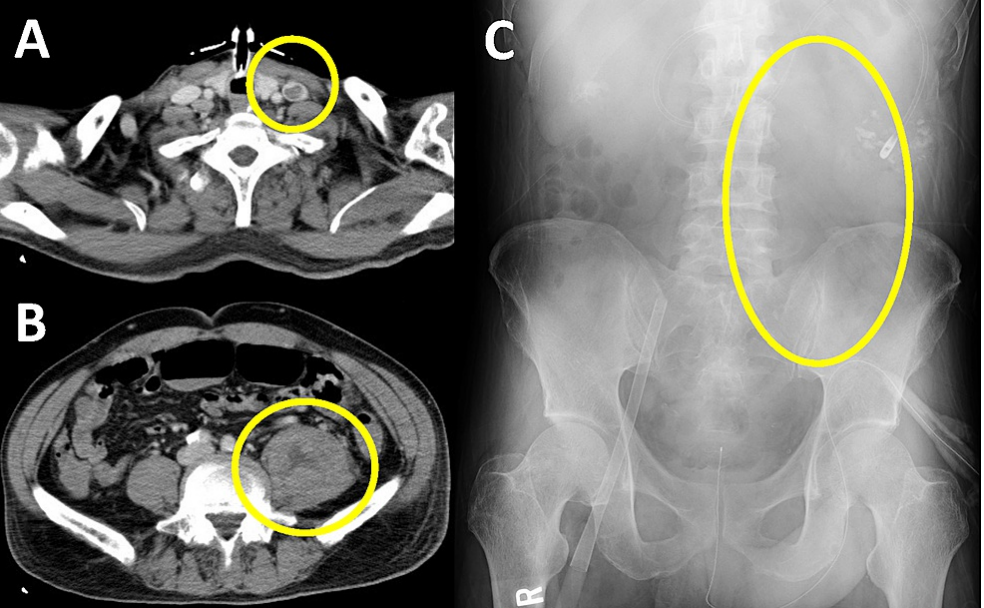
Computed tomography on day 20 and abdominal X-ray on day 14 (A) Enhanced computed tomography on day 20 showed left internal jugular thrombosis. (B) No extravasation was found in the left iliopsoas hematoma by the contrast media. (C) Abdominal X-ray on day 14 showed the appearance of the left psoas sign, suggesting correspondence to iliopsoas hemorrhage.

**Figure 3:**
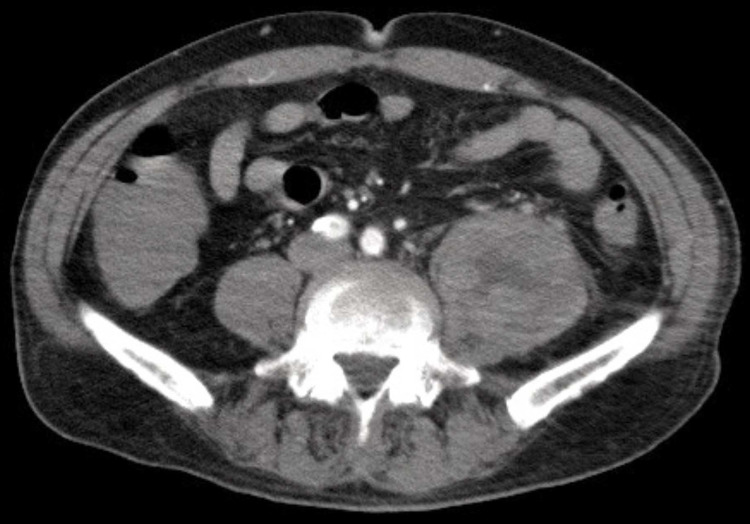
Computed tomography on day 26 Follow-up enhanced computed tomography on day 26 revealed no apparent progression of left iliopsoas hematoma, and no extravasation of contrast media was seen.

On day 20, anemia progressed, and the patient experienced pain when his left lower limb was passively mobilized for routine postural change. To discern internal hemorrhagic conditions, he underwent a whole-body CT scan with contrast media, which revealed left-sided psoas hematoma and left internal jugular vein thrombosis. No active extravasation of contrast media was observed, but a retrospective review of abdominal radiographs on day 14 suggested the appearance of the left psoas sign while the patient was under ECMO management (shown in Figure 2). The follow-up CT scan on day 26 showed no progression of the hematoma (Figure 3), and there was no evidence of active bleeding within the laboratory data or hemodynamic stability. We initiated intravenous heparinization to prevent jugular vein thrombosis. On day 31, mechanical ventilation required minimal support, and he was transferred back to another hospital for additional care.

## Discussion

This case involved a middle-aged Japanese man with type 1 diabetes mellitus and a smoking habit who developed severe COVID-19 pneumonia. He underwent VV-ECMO management with conservative anticoagulation, which complicated the iliopsoas hematoma. We detected IPH retrospectively by abdominal radiography and successfully treated the patient with conservative anticoagulation. These strategies might be effective for other Japanese, possibly Asian patients, as well.

Hospitalized COVID-19 patients have a higher incidence of co-morbidities such as hypertension, diabetes, and cardiovascular diseases when compared to non-hospitalized COVID-19 patients [4]. Severe COVID-19 pneumonia can be managed with VV-ECMO [5]. ECMO requires anticoagulation therapy to maintain the circuit, which is often challenging. Some institutions monitor aPTT and activated clotting time (ACT) to guide anticoagulation, but the two results are poorly correlated. Hemostatic differences between Asians and Caucasians have been reported, suggesting a higher bleeding tendency in the former and an increased risk of thrombotic events in the latter [6]. The difference may be related to the consumption of fish in the daily diet [7]. The evidence suggests the importance of establishing anticoagulation strategies within individual populations, which take into account factors such as race.

IPH is defined as spontaneous retroperitoneal hemorrhage (SRH), which is defined as bleeding within the retroperitoneal space without associated trauma, iatrogenic manipulation, or aortic aneurysm [8]. In previous reports, 10.1% of all SRHs with various origins were initially misdiagnosed [8]. The incidence of IPH during an intensive care unit stay was reported to be 3.8 cases/1,000 admissions and was associated with elevated mortality [9]. IPH has also been reported in patients undergoing ECMO [3], but its precise epidemiological nature remains unknown. The suggested risk factors of IPH include aging, obesity, anticoagulation, hemodialysis, mobilization to a wheelchair, physical therapy, and anticoagulation therapy [9].

In this case, the development of IPH occurred days after ECMO initiation and seemed unrelated to cannulation. Symptoms of IPH are nonspecific and atypical, which makes diagnosis difficult. Mechanical stress and prolonged supine positioning may be associated with IPH development. More than half of IPH cases require invasive procedures to achieve hemostasis and stabilization [9]. In our case, we managed the problem using conservative treatment. There is a possibility that the hemorrhage that occurred was related not only to heparin administration but also to the characteristics of a Japanese individual. The hypercoagulative state caused by COVID-19 may have improved hemostasis without requiring an invasive approach.

At the same time, an enhanced CT scan revealed left jugular vein thrombosis at which time a central venous catheter (CVC) was placed. Although CVC itself is a risk factor for venothrombosis, it may have developed throughout the same period as the iliopsoas hemorrhage. Taking this into consideration, there may be an extremely narrow therapeutic range for anticoagulation to prevent clot formation within the ECMO circuit that will not produce hemorrhagic events. We should stay alert to hemorrhagic complications, including IPH, by keeping an eye on clues such as circulatory instability, anemia, pain associated with limb movements, and abnormalities on abdominal X-rays such as psoas signs.

## Conclusions

We report a case of IPH that could have been identified earlier by radiography on conservative anticoagulation in a patient with severe COVID-19 during ECMO management. For Japanese or Asian patients with severe COVID-19 pneumonia who undergo VV-ECMO, monitoring for IPH is important, especially when progressive anemia and lower limb pain are observed.
